# Identification of *Plasmodium* dipeptidyl aminopeptidase allosteric inhibitors by high throughput screening

**DOI:** 10.1371/journal.pone.0226270

**Published:** 2019-12-18

**Authors:** Mateo I. Sanchez, Laura E. de Vries, Christine Lehmann, Jeong T. Lee, Kenny K. Ang, Christopher Wilson, Steven Chen, Michelle R. Arkin, Matthew Bogyo, Edgar Deu

**Affiliations:** 1 Departments of Pathology and Microbiology & Immunology, Stanford School of Medicine, Stanford, CA, United States of America; 2 Chemical Biology Approaches to Malaria Lab, The Francis Crick Institute, London, United Kingdom; 3 Small Molecule Discovery Center and Department of Pharmaceutical Chemistry, University of California at San Francisco, San Francisco, CA, United States of America; Instituto Rene Rachou, BRAZIL

## Abstract

Dipeptidyl aminopeptidases (DPAPs) are cysteine proteases that cleave dipeptides from the N-terminus of protein substrates and have been shown to play important roles in many pathologies including parasitic diseases such as malaria, toxoplasmosis and Chagas’s disease. Inhibitors of the mammalian homologue cathepsin C have been used in clinical trials as potential drugs to treat chronic inflammatory disorders, thus proving that these enzymes are druggable. In *Plasmodium* species, DPAPs play important functions at different stages of parasite development, thus making them potential antimalarial targets. Most DPAP inhibitors developed to date are peptide-based or peptidomimetic competitive inhibitors. Here, we used a high throughput screening approach to identify novel inhibitor scaffolds that block the activity of *Plasmodium falciparum* DPAP1. Most of the hits identified in this screen also inhibit *Plasmodium falciparum* DPAP3, cathepsin C, and to a lesser extent other malarial clan CA proteases, indicating that these might be general DPAP inhibitors. Interestingly, our mechanism of inhibition studies indicate that most hits are allosteric inhibitors, which opens a completely new strategy to inhibit these enzymes, study their biological function, and potentially develop new inhibitors as starting points for drug development.

## Introduction

Malaria is a devastating infectious disease caused by parasites of the *Plasmodium* genus. With half of the world population at risk, over 200 million clinical cases per year, and half a million deaths, malaria remains one of the major global health burdens[1]. Malaria is transmitted through the bite of *Anopheles* mosquitoes. Parasites first establish an asymptomatic infection in the liver where they replicate within hepatocytes. After being released into the blood stream, they multiply exponentially through multiple rounds of red blood cell (RBC) invasion, asexual replication, and egress from infected RBCs (iRBCs). A small portion of circulating parasites develops into male and female gametocytes, which reproduce sexually in the mosquito midgut after being ingested during a blood meal. Parasites then cross the midgut epithelial, multiply, and travel to the salivary glands from where they are transmitted to the next human host. The exponential asexual replication of parasites during the erythrocytic cycle is responsible for all the symptoms and pathology of malaria, and the stage at which parasites are more likely to become drug resistance.

*Plasmodium* parasites have become resistant to most front-line drugs, and resistance to new treatments such as artemisinin-based combination therapy is quickly emerging[2], thus making the identification of novel antimalarial targets extremely urgent[3,4]. Proteases are proven therapeutic targets for a variety of pathologies, including infectious diseases such as AIDS or hepatitis C[5,6]. More importantly, proteases play essential roles at all stages of parasite development[7]. In particular, dipeptidyl aminopeptidases (DPAPs) have been shown to be important during the sexual[8,9] and asexual stages of parasite development[10–12], thus making them potential drug targets to treat malaria and prevent its transmission. These clan CA cysteine proteases cleave dipeptides from the N-terminal of substrate proteins[13,14]. In addition, DPAPs have been shown to be druggable targets. The mammalian homologue cathepsin C (CatC) has been pursued by the pharmaceutical industry[15–18] for the treatment of chronic inflammatory diseases due to its role in activating proinflammatory serine proteases, such as neutrophil elastase, granzyme A and B, or cathepsin G[19–22]. Recently, highly specific CatC inhibitors from GlaxoSmithKline (GSK2793660)[23] and AstraZeneca (AZD-7986)[24] have been studied in phase I clinical trials.

Three DPAPs are conserved in *Plasmodium* species. In iRBCs, DPAP1 localizes in the digestive vacuole where it has been proposed to play an essential role in the later stages of the hemoglobin degradation pathway[10]. This catabolic proteolytic pathway provides free amino acids for protein synthesis and liberates space within the iRBC to allow parasite growth. However, the importance of DPAP1 in this pathway has not been validated genetically. DPAP2 is only expressed in gametocytes and its knock out (KO) results in a significant reduction of gamete egress from iRBCs[9]. Finally, using a conditional knockout approach we have recently shown that DPAP3 activity is critical for efficient RBC invasion[12]. Multiple attempts to directly KO DPAP1 or DPAP3 in *P*. *falciparum* have been unsuccessful strongly indicating that these proteases are important during the erythrocytic cycle[10,12]. However, in the *P*. *berghei* murine model of malaria, KO of DPAP1 or DPAP3 results in a significant delay in asexual replication[25–27], thus suggesting that DPAPs are important but not essential in *P*. *berghei*. Overall, very little is known about the molecular function of *Plasmodium* DPAPs, nor whether they might perform redundant functions. Therefore, specific inhibitors of these proteases will be very valuable tools to study their biological function and to validate their potential as antimalarial targets.

While we have been able to develop potent DPAP covalent inhibitors, stability, off-target effects, and/or toxicity issues have prevented us from robustly validate these targets in murine models of malaria[11]. In an attempt to identify novel DPAP inhibitor scaffolds, we pursued a high throughput screening (HTS) approach for which we developed an assay to specifically measure DPAP1 activity in parasite lysates. This assay uses the (PR)_2_Rho substrate, which we have shown is exclusively cleaved by DPAP1 in trophozoite lysates[28]. This assay precludes the need to purify or express DPAP1 and allows the measurement of its activity within a more biologically relevant environment.

In this study we present the results of this HTS campaign where we identified over one hundred active compounds from a library of more than 100,000 small drug-like molecules. Follow-up studies were performed on 33 hits, including mechanism of inhibition studies for the most potent inhibitors. Most compounds identified in this study also block the activities of DPAP3, CatC, and to a lesser extend of falcipains 2 and 3 (FP2 and FP3), thus validating these new compounds as genuine inhibitors of clan CA proteases. FP2 and FP3 are *Plasmodium* endopeptidases at the top of the proteolytic pathway that degrades hemoglobin in the digestive vacuole. Interestingly, most inhibitors do no inhibit DPAP1 or CatC through a competitive inhibition model but rather through a partial competitive or partial mixed inhibition model, which suggests the presence of allosteric regulatory sites.

## Materials and methods

### Materials and reagents

The synthesis and characterization of the fluorogenic substrates (PR)_2_Rho[28], VR-ACC[29], and nVal-nLeu(o-Bzl)-ACC[30] have been previously described. Z-LR-AMC and GR-AMC were purchased from Sigma. All DPAPs were purified as previously described: DPAP1 was purified from parasite lysates[29], and bovine CatC from spleen homogenates[31,32]; recombinant DPAP3 was expressed in insect cells and purified from culture supernatants[12]. Recombinant FP2 and FP3 were a kind gift from Prof. Phillip Rosenthal (UCSF). Hit compounds selected for follow-up studies were purchased from Vitas M. Lab, Chem Div, SPECS, and ChemBridge (catalogue numbers are reported in S1 Table).

### HTS assay, cherry-picking, and hit validation

HTS was performed in black 384-well plates. The first and last two columns of each plate contained our negative (DMSO) and positive (10 μM JCP410) controls, and the 20 central columns the test compounds at 10 μM. Compounds were first diluted 50-fold into assay buffer (50 mM sodium acetate, 5 mM MgCl_2_, 10 mM β-mercaptoethanol, and 0.1% CHAPS, pH 5.5) from a 10 mM DMSO stock plates. One microliter of this 200 μM intermediate stock plate was dispensed into the assay plate. Reaction was initiated with 10 μL of a 20 μM stock of (PR)_2_Rho in assay buffer, followed by 10 μL of trophozoite lysates diluted 1:50 in assay buffer. After 30 min, the reaction was quenched with 20 μL of 0.5 M acetic acid, and the fluorescent intensity measured at 523 nm (λ_ex_ = 492 nm) using an Analyst HT multimode plate reader (Molecular Devices). Test compounds were transferred to the intermediate stock plates and assay plates with a BioMek FXP automated liquid handler (Beckman Coulter). Assay reagents were added using a Matrix Wellmate bulk dispenser (Thermo Scientific).

Z’ values were calculated for each of the 333 plates used to screen the library. 227 compounds (> 50% inhibition) were cherry-picked from the 10 mM stock plates, and DPAP1 inhibition confirmed in a dose dependent manner using the same end-point assay. Thirty-seven compounds were purchased for follow-up studies. To confirm that the purchased compounds were indeed DPAP1 inhibitors, dose response studies were performed at 10 μM (PR)_2_Rho both in parasite lysates and with purified DPAP1 but using a continuous assay rather than an end-point assay in a SpectraMax 5e (Molecular Devices) multimode platereader. Purified DPAP1 was used at 1 nM.

### Dose response inhibition assays

Each protease was used at 1 nM in the HTS assay buffer described above. Dose response inhibition studies to obtained IC_50_ values for each of the proteases tested were performed using fluorogenic substrates at the *K*_m_ concentrations determined under our assay conditions: VR-ACC for DPAP1 (*K*_m,DPAP1_ = 20 μM)[29], nVal-nLeu(o-Bzl)-ACC for DPAP3 (*K*_m,DPAP3_ = 1.4 μM)[30], GR-AMC for CatC (*K*_m,CatC_ = 40 μM), and Z-LR-AMC for the FPs (*K*_m,FP2_ = 5 μM; *K*_m,FP3_ = 20 μM). For all assays, substrate turnover was measured for 30 min at 460 nm (λ_ex_ = 355nm) using a SpectraMax M5e (Molecular Devices) multimode plate reader. Each dose response was performed in triplicate with inhibitor concentrations ranging between 0.01 and 50 to 200 μM depending on compound solubility. Initial velocity as a function of inhibitor concentration was fit to Eq. 1 in GraphPad Prism to obtain IC_50_ values.

$$\frac{V_{i}}{V_{0}}=(1-\Delta )+\frac{\Delta }{1+{\left(\frac{{IC}_{50}}{\left[I\right]}\right)}^{HC}}$$ Eq 1

*V*_i_ and *V*_0_ are the initial velocities in the presence or absence of inhibitor, respectively, Δ, the maximum inhibition fraction, and HC, the Hill coefficient. Note that the maximum % inhibition is equal to 100 x Δ.

### Mechanism of inhibition studies

Mechanism of inhibition (MOI) studies were performed in 384-well black plates. For each compound and enzyme, we measured a matrix of initial velocities using 4–6 concentrations of substrate (0.25–8 x *K*_m_) by 6–8 concentrations of inhibitor (0–100 μM). At each inhibitor concentration the dependence of initial substrate turnover on substrate concentration was fitted to the Michaelis Menten equation to obtain *K*_m,app_ and *V*_max,app_. The dependency of these parameters on inhibitor concentration was examined to select the most appropriate inhibition model. Initial velocities as a function of substrate and inhibitor concentrations were globally fitted to the chosen MOI model using GraphPad Prism. The kinetic and inhibition parameters from these global curve fits were then used to predict the dependence of *K*_m,app_ and *V*_max,app_ on inhibitor concentration. This allowed us to visualize how well this predicted *K*_m,app_ and *V*_max,app_ values matched those independently determined at each inhibitor concentration. To statistically validate the chosen inhibition model, we performed F-test null-hypothesis tests in GraphPad Prism.

### Tryptophan fluorescence studies

CatC (2 μM) was incubated for 30 min in assay buffer with DMSO or 20 μM of JCP410, a very potent covalent inhibitor of CatC (*k*_i_/*K*_i_ = 5.8x10^6^ M^-1^s^-1^)[30]. The enzyme was then diluted 2-fold in assay buffer containing different concentrations of inhibitors. Emission spectra were measured between 300–400 nm (λ_ex_ = 280 nm) using a SpectraMax M5e multimode plate reader (Molecular Devices) in black 96-well plates. Fluorescence intensity at the maximum emission wavelength (335 nm) was plotted as a function of inhibitor concentration.

### Parasite culture and replication assay

Anonymized human blood used to culture malaria parasites was sourced from the United Kingdom National Health Service Blood and Transplant Special Health Authority. No ethical approval is required for its use. *P*. *falciparum* D10 parasites were cultured in RPMI-based media supplemented with Albumax as previously described[33]. Parasite cultures were synchronized by treating ring stage parasites with sorbitol every 48 h. Trophozoite pellets were obtained by collecting iRBCs at 24–30 h post invasion, lysing the RBC and parasitophourous vacuole membranes with saponin, and storing the parasite pellets at -80°C. These were then lysed by adding 2 volumes of 1% NP40 in PBS and incubating the samples in ice for 1 h. The soluble fraction was collected after a 10 min spin at 13,000 rpm in a microcentrifuge, frozen in liquid N_2_, and stored at -80°C. For replication assays, ring stage parasites at 1% parasitemia and 1% hematocrit were treated with different concentrations of compound for 72 h. After fixation with 0.01% glutaraldehyde, samples were permeabilized for 10 min with 0.1% Triton X, and DNA stained with propidium iodide. The percentage of iRBCs in each sample was quantified by FACS as previously described[11]. Three biological replicates were performed for each compound.

### Cytotoxicity assay

Human foreskin fibroblasts were cultured in flat bottom 96-well plates and were treated for 24h one day before they reached confluency with different concentrations of compound. Cell viability was measured using the Promega CellTiter-Glo Assay using the manufacturer’s instructions. Cytotoxicity EC_50_ values were estimated by fitting the data to a dose response curve. Four biological replicates were performed for this experiment.

## Results

### HTS against DPAP1 in parasite lysates

**Fig 1:**
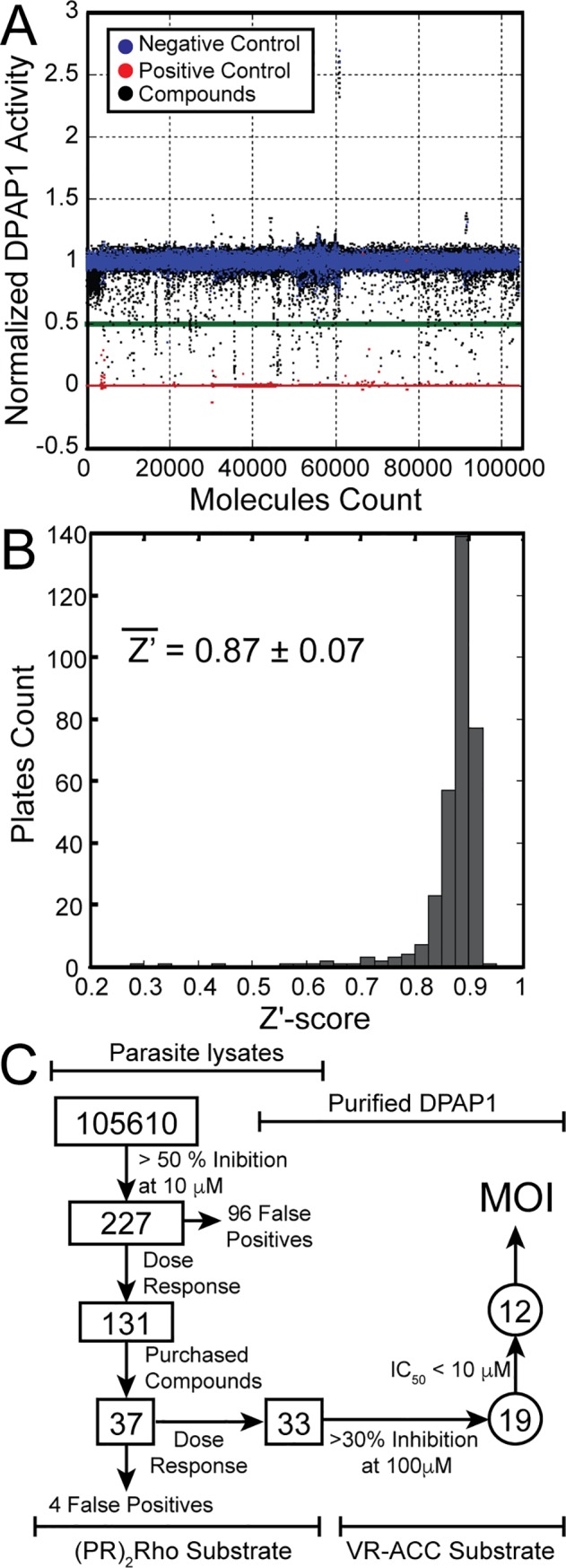
DPAP1 HTS and triage. **(A**) Screening results. More than 100,000 small molecules were screened at 10 μM in parasite lysates. DPAP1 activity was measured using the DPAP1-specific fluorogenic substrate (PR)_2_Rho in an end-point assay. Normalized activity is shown for each compound (black) and for the positive (10 μM JCP410, red) and negative controls (DMSO, blue). The green line indicates the 50% inhibition cut-off that was applied to cherry-pick individual compounds for further studies. (**B**) HTS performance. Distribution of Z’-values for the 333 plates used in the screen is shown for an interval of 0.025. (**C**) Triage process. Our initial screen in parasite lysates identified 227 compounds that inhibited DPAP1 by more than 50% at 10 μM. 131 showed a dose response behavior between 0.02 and 50 μM using the same end-point assay. 37 compounds were purchased for further studies. Four were found to be false positives while the remaining 33 inhibited purified DPAP1 in a dose-dependent manner using the (PR)_2_Rho substrate both in parasite lysates and against purified DPAP1. Compounds that inhibited purified DPAP1 by more than 30% at 100 μM using the VR-ACC substrate were studied in detail, and the mechanism of inhibition (MOI) determined for 12 of them.

We screen two different libraries of compounds: the UCSF Small Molecule Discovery Center’s Diversity Collection, which is composed of 104,121 compounds and was assembled from numerous commercial vendors (ChemBridge, ChemDiv and SPECS), and the Celera Protease Inhibitor Collection of 1,817 compounds, which is unique and not commercially available. The latter is composed entirely of compounds synthesized by medicinal chemists at Celera Genomics in the course of various discovery campaigns targeting human cathepsins, primarily cathepsins K, S, and B. A total of 105,938 small molecules were screened at 10 μM in 384-well plates using an endpoint assay. Briefly, parasite lysates were diluted in assay buffer and incubated with a mixture of compound and (PR)_2_Rho for 30 min before quenching the reaction with acetic acid. The level of substrate cleavage was measured by fluorescence at 523 nm (λ_ex_ = 492 nm). JCP410, a well-characterized vinyl sulfone covalent inhibitor of DPAP1[33], and DMSO were used as positive and negative controls, respectively. Normalized DPAP1 activity for the full screen and the distribution of Z’-values across all plates are shown in Fig 1A and 1B, respectively. The average Z’-value for the full screen was 0.87, which attests to the robustness of the assay. The HTS results and the compounds structures are reported in S2 Table. Overall, we identified 1077 compounds that significantly decreased DPAP1 activity, i.e. more than three standard deviations below the average DMSO control activity value (> 20% inhibition). The 227 most active compounds (> 50% inhibition at 10 μM) were cherry-picked for validation studies.

Selected compounds were tested between 0.02 and 50 μM using the same endpoint assay used in the initial screen. Fifty-eight percent showed a concentration dependent inhibition effect and were considered validated hits (Fig 1C). Thirty seven were purchased for follow-up studies based on their potency, structural diversity, and commercial availability (S1 Table): 32 correspond to hit compounds identified in the initial screen, 2 are analogues of validated hits that were no longer commercially available (SMDC153437A and SMDC168384A), and 3 are structurally related to a family of compounds that showed a very high hit-rate during the screen (SMDC170123, SMDC170136, SMDC170156), namely 3,8-disubstituted 6-oxo-pyridothialazine-9-carbonitriles (DOPTACN). Note that 70 DOPTACN compounds were present in the HTS library; 61 significantly inhibited DPAP1 (> 20% inhibition), and 27 decreased its activity by more than 50% (S3 Table).

### Validation of active compounds

To validate that the purchased compounds are DPAP1 inhibitors, they were tested between 0.01 and 100 μM both in trophozoite lysates or with purified DPAP1 using 10 μM (PR)_2_Rho in a continuous rather than an end-point assay. Four of the 37 purchased compounds were not able to inhibit DPAP1 at 100 μM and were considered false positives (Fig 1C and S1 Table).

### Characterization of DPAP1 inhibitor hits

**Fig 2:**
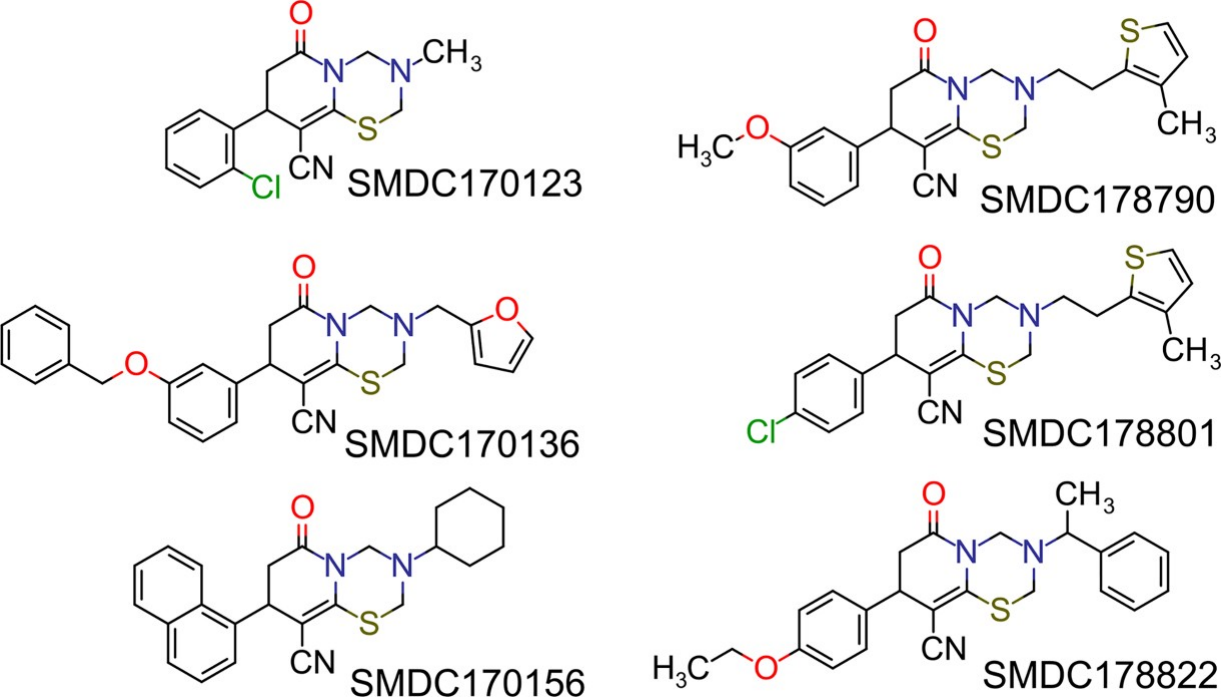
Structure of DOPTACN hits. The structure of the compounds reported in Table 1 are shown.

**Fig 3:**
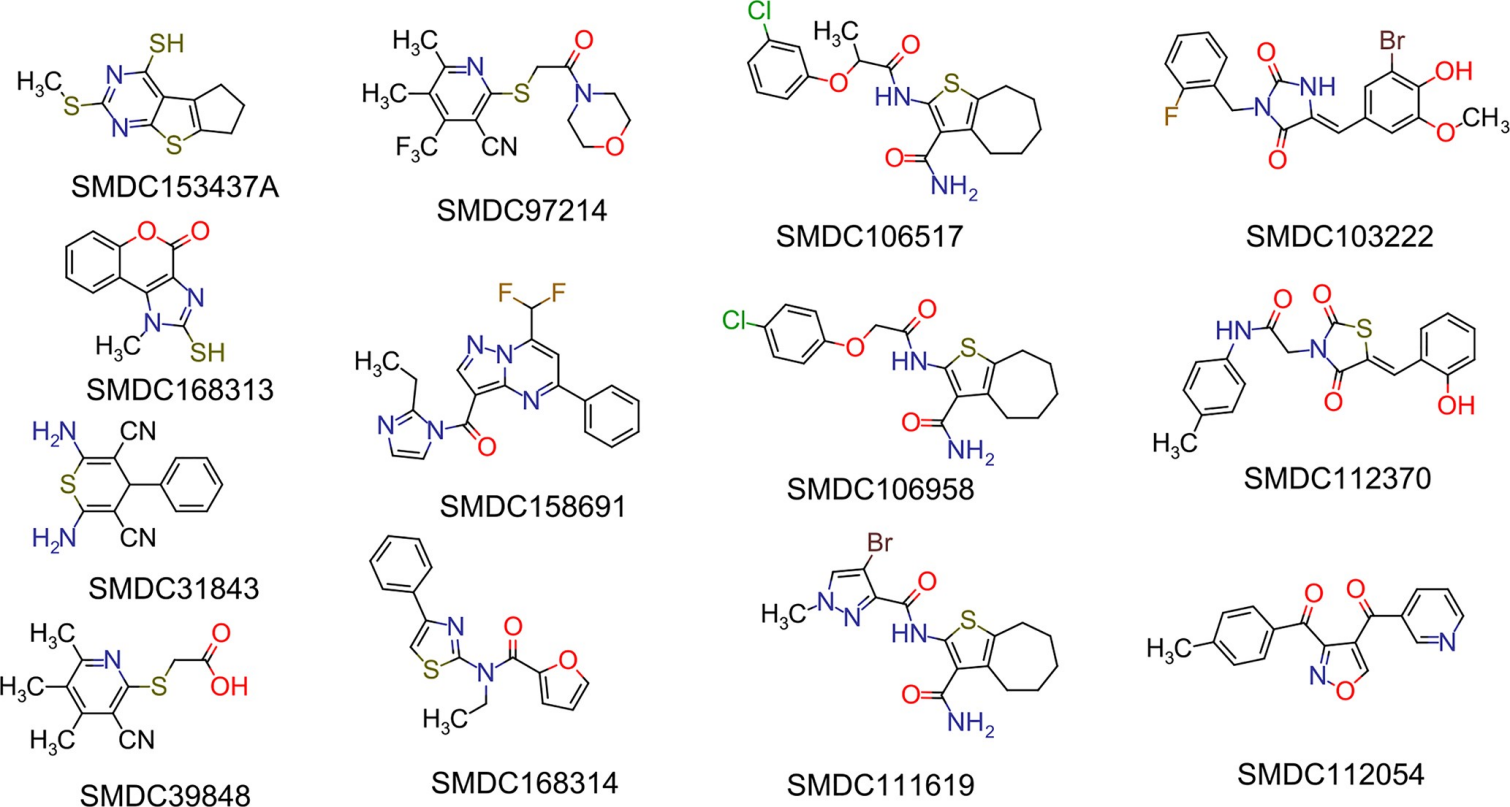
Structure of other validated hit compounds. The structure of the compounds reported in Table 2.

**Fig 4:**
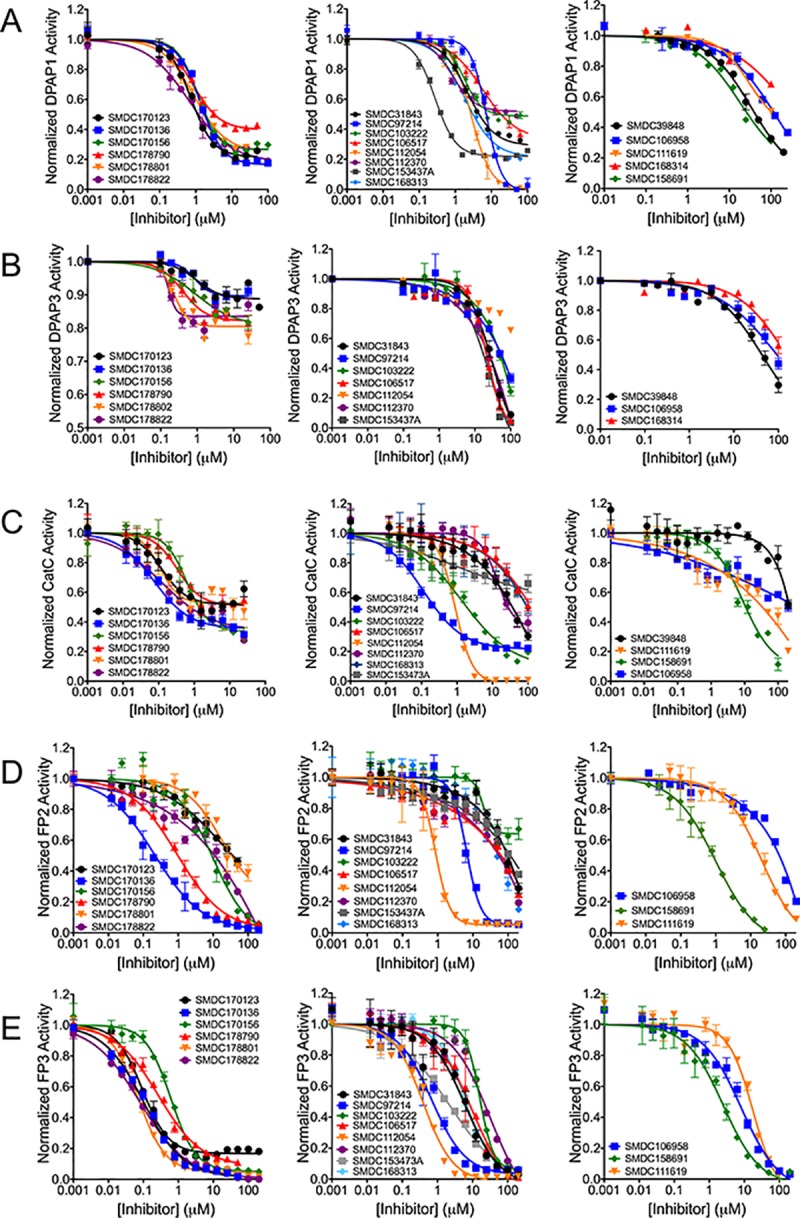
Dose response inhibition curves for hit compounds. The inhibitory effect of purchased compounds against DPAP1 (**A**), DPAP3 (**B**), CatC (**C**), FP2 (**D**) or FP3 (**E**) was tested at different compound concentrations using different fluorogenic substrates. Substrates were used at the *K*_m_ concentration of each enzyme to obtain comparable IC_50_ values. Protease activity was normalized to the DMSO control. Each dose-response was fitted to Eq 1. Structurally related DOPTACN compounds are shown on the left graphs. Compounds for which solubility issues prevented us from obtaining a maximum inhibition baseline for DPAP1 are show on the right graphs. The remaining compounds are presented in the middle graphs. IC_50_ and maximum percentage inhibition values are reported in Tables 1 and 2. All dose-response were performed in triplicate. Error bars represent standard errors.

(PR)_2_Rho is not an optimal substrate to determine accurate inhibition constants (*K*_i_) because it needs to be cleaved twice to release the highly fluorescent rhodamine group^28^. Therefore, its turnover by DPAP1 does not follow classical Michaelis Menten behavior. Instead, we used the VR-ACC DPAP substrate[29] for follow-up studies using purified DPAP1. The 33 validated inhibitors were first tested at 100 μM against DPAP1 using *K*_m_ concentrations of VR-ACC (20 μM). IC_50_ values were measured for the 19 most potent inhibitors (> 30% inhibition at 100 μM). Six of these are DOPTACN compounds, the structure of which are shown in Fig 2. The structure of the remaining hit compounds are shown in Fig 3. Interestingly, most compounds were not able to fully inhibit DPAP1, and the maximum level of DPAP1 inhibition ranged between 40 and 100% (Fig 4A and Tables 1 and 2). This partial inhibition behavior indicates that these molecules do not bind at the same site as the substrate.

**Table 1:** IC_50_ and EC_50_ values for DOPTACN hit compounds.

SMDC#	IC_50_ (μM) & Maximum % Inhibition^*a*^					*Pf* EC_50_ (μM)^*b*^ & % Inh @ 100 μM	Tox EC_50_ (μM)^c^ & S.I.
	DPAP1	DPAP3	CatC	FP2	FP3		
170123	0.74 (0.05) 78 (2)	1.0 (0.3) 11 (2)	0.12 (0.03) 48 (3)	~35 (4) >50	0.093 (0.007) 83 (4)	>100 64 (9)	N.D.
170136	1.3 (0.1) 83 (2)	0.9 (0.2) 10 (1)	0.07 (0.01) 65 (2)	0.21 (0.02) 98 (2)	0.10 (0.01) 99 (3)	14 (2) 79 (3)	180 (20) 13
170156	1.1 (0.1) 73 (2)	0.9 (0.3) 19 (2)	0.62 (0.07) 65 (2)	9.0 (1.5) 100 (10)	0.61 (0.04) 95 (5)	31 (6) 66 (3)	240 (50) 8
178790	0.84 (0.07) 60 (2)	0.40 (0.08) 18 (1)	0.33 (0.06) 66 (3)	0.9 (0.1) 98 (2)	0.27 (0.04) 92 (4)	4 (2) 74 (5)	200 (50) 50
178801	0.9 (0.1) 75 (3)	0.23 (0.04) 20 (1)	0.11 (0.02) 48 (2)	17 (10) 80 (15)	0.069 (0.004) 93 (4)	31 (7) 78 (2)	52 (9) 1.7
178822	0.65 (0.07) 82 (2)	0.16 (0.03) 16 (1)	0.12 (0.02) 48 (3)	12 (4) 100 (8)	0.080 (0.006) 99 (1)	28 (6) 78 (5)	100 (25) 3.6

^*a*^ When we could not obtain a maximum inhibition baseline, an approximate IC_50_ value is given (~ sign) as the concentration where we observed 50% inhibition. The maximum level of inhibition obtained at the highest concentration is also reported (> sign).

^*b*^ *In vitro antiparasitic* activity against *P*. *falciparum* was determined using a standard 72h replication assay (3 biological replicates).

^*c*^ Cytotoxicity EC_50_ values were determined in human foreskin fibroblast cells using the CellTiter-Glo Assay from Promega (4 biological replicates). The specificity index (S.I.) was calculated by dividing the cytotoxicity EC_50_ value by the *P*. *falciparum* antiparasitic EC_50_ value.

All experiments were performed in triplicate. Standard errors are shown in parentheses.

**Table 2:** IC_50_ and EC_50_ values for hit compounds.

SMDC #	IC_50_ (μM) & Maximum % Inhibition^*a*^					*Pf* EC_50_ (μM)^*b*^ & % Inh @ 100 μM	Cytox EC_50_ (μM) ^*c*^ & S.I.
	DPAP1	DPAP3	CatC	FP2	FP3		
153437A	0.27 (0.02) 78 (1)	25 (7) 100 (15)	1.2 (1.5) 47 (8)	~100 >60	2.3 (0.5) 100 (4)	N.D. 28 (2)	N.D.
168313	1.8 (0.2) 78 (2)	N.I.	~100 >50	~50 >85	6.3 (0.5) 100 (7)	N.D. 20 (1)	N.D.
31843^*PAIN*^	2.2 (0.2) 71 (2)	30 (8) 100 (14)	~25 >75	~100 >70	5.7 (0.8) 100 (4)	N.D. 24 (3)	N.D.
39848	34 (7) 94 (6)	~100 >50	~100 >50	N.I.	N.I.	N.D. 13 (1)	N.D.
97214	7.2 (0.5) 100 (3)	~100 >70	0.11 (0.01) 78 (1)	0.25 (0.02) 96 (2)	0.68 (0.08) 97 (3)	N.D. 32 (4)	N.D.
158691	18 (8) 88 (12)	N.I.	9(3) 91 (9)	1.00 (0.03) 100 (3)	2.50 (0.04) 100 (4)	9 (1) 88 (3)	70 (8) 8
168314	200 (80) 100	~100 >50	N.I.	N.I.	N.I.	N.D. 35 (5)	N.D.
106517	5.7 (0.6) 67 (2)	21 (3) 100 (8)	~100 >50	~100 >80	7.2 (0.8) 100 (8)	15 (3) 83 (5)	160 (40) 11
106958	80 (50) 85 (2)	~100 >50	~100 >50	~100 >80	6.5 (0.6) 100 (5)	N.D. 25 (2)	N.D.
111619	~ 100 > 50	N.I.	~50 >80	21 (5) 100 (7)	18 (5) 100 (10)	N.D. 35 (4)	N.D.
103222	1.7 (0.3) 51 (2)	~50 >75	1.4 (0.3) 91 (4)	20 (3) 36 (3)	15.9 (0.8) 94 (1)	23 (8) 75 (2)	400 (120) 17
112370	0.6 (0.2) 42 (3)	31 (6) 100 (10)	13.5 (1.5) 64 (3)	~50 >80	21 (3) 100 (6)	N.D. 6 (1)	N.D.
112054	2.7 (0.1) 100 (1)	>100 >35	0.9 (0.1) 100 (3)	0.95 (0.02) 95 (2)	0.52 (0.04) 100 (2)	35 (5) 83 (9)	350 (100) 10

^*a*^ When we could not obtain a maximum inhibition baseline, an approximate IC_50_ value is given (~ sign) as the concentration where we observed 50% inhibition. The maximum level of inhibition obtained at the highest concentration is also reported (> sign).

^*b*^ *In vitro antiparasitic* activity against *P*. *falciparum* was determined using a standard 72h replication assay (3 biological replicates).

^*c*^ Cytotoxicity EC_50_ values were determined in human foreskin fibroblast cells using the CellTiter-Glo Assay from Promega (4 biological replicates). The specificity index (S.I.) was calculated by dividing the cytotoxicity EC_50_ value by the *P*. *falciparum* antiparasitic EC_50_ value.

^*PAIN*^ Only one hit compound in which we performed follow-up study (SMDC31843) was identified as a PAIN molecule.

All experiments were performed in triplicate. Standard errors are shown in parentheses.

N.I. No inhibition. N.D. Not determined because treatment at 100 μM show no significant decrease in parasitemia.

### Some DPAP1 inhibitors block parasite replication

The antimalarial activity of the 19 most potent DPAP1 inhibitors was first tested at 100 μM using a standard 72 h parasite replication assay against *P*. *falciparum* (Tables 1 and 2). Ten compounds decreased parasitemia by more than 50% and were further tested in a dose-dependent manner (S1 Fig). The EC_50_ for most of these compounds ranged between 4 and 35 μM (Tables 1 and 2). However, there is no clear correlation between IC_50_ and EC_50_ values. In order to inhibit DPAP1, compounds have to cross four membranes (RBC, parasitophorous vacuole, cytosolic, and digestive vacuole membranes). Also, sustained inhibition of DPAP1 for several hours is likely necessary to block parasite replication[11]. Therefore, differences in cell permeability and compound stability might account for this lack of correlation. Further studies will be necessary to determine whether the antimalarial properties of these compounds are due to DPAP1 inhibition, inhibition of other clan CA proteases, or other off-target effects. However, we did not think that the antiparasitic activity of these compounds is due to general toxicity since none of them kill mammalian cell at 10 μM (S2 Table).

To test this hypothesis we performed cell viability assays in human foreskin fibroblast (HHF) using the CellTiter-Glo Assay (Promega) that measures ATP content within cells. HFF cells were treated for 24h with different concentrations of compounds (0.2–200 μM), those for which antiparasitic EC_50_ values could be obtained (9 compounds). Four biological replicates were performed, and the data was fitted to a dose response curve (S2 Fig) to obtain cytotoxicity EC_50_ values (reported in Tables 1 and 2). For all compounds, the toxicity EC_50_ values were above 50 μM, and for most of them the specificity index (measured as the ratio between the cytotoxicity and antiparasitic EC_50_ values) was above 10, with SMDC178790 having the highest specificity index (S.I. = 50). Overall, all compounds are more potent at killing *P*. *falciparum* parasites than human cells. However, the fact that they all kill human cells at high micromolar concentrations suggests that their antiparasitic activity might also be due to some general cytotoxic effects. That said, as is shown below, most DPAP inhibitors identified in this screen inhibit other clan CA malarial proteases (falcipains 2 and 3), as well as mammalian CatC. Therefore, the cytotoxicity might also be due to inhibition of multiple human Clan CA proteases in HFFs. Overall, these results indicate that much more potent and DPAP specific inhibitors need to be developed from these inhibitor scaffolds to rule out general cytotoxic effects and to validate that their antiparasitic activities are mediated through DPAPs inhibition.

### Selectivity of DPAP1 inhibitors against other Cys proteases

To determine the specificity of the 19 selected DPAP1 inhibitors against other cysteine proteases, we tested them against DPAP3, bovine CatC, FP2 and FP3 using different fluorogenic substrates (Fig 4B–4E). In order to obtain comparable IC_50_ values, the substrate concentration was fixed to the *K*_m_ value determined under our assay conditions for each protease:substrate pair. All IC_50_ and maximum percentage inhibition values are reported in Tables 1 and 2. Most compounds were able to inhibit all proteases, but we observed significant differences in potency (IC_50_) and maximum level of inhibition between the different proteases. This indicates that the observed partial inhibition effects are not due to compound concentration effects such as aggregation, instability, or assay interference. Note that only one of the hit compounds, SMDC31843, was identified as a potential pan-assay interference (PAIN) compound[34]. Also, none of the hit compounds was able to inhibit caspase 2 or 6 by more than 50% at 10 μM, as determined in previous HTS campaigns performed at the SMDC (S1 Table). Overall, this suggests that the compounds identified in this screen are clan CA protease inhibitors rather than highly promiscuous compounds.

Based on previous screens of peptidic or peptidomimetic inhibitors, it has been relatively difficult to identify compounds that discriminate between DPAP1 and DPAP3 or between FP2 and FP3[11,33]. However, most DPAP1 inhibitors identified in this screen are relatively poor DPAP3 inhibitors (Fig 4A and 4B and Tables 1 and 2). For example the DOPTACN compound achieve higher levels of maximum inhibition for DPAP1 (60–83%) than for DPAP3 (10–20%), and SMDC153437A and SMDC168313 are at least 100-fold more potent for DPAP1 than DPAP3 (IC_50,DPAP1_ = 0.27 μM vs. IC_50,DPAP3_ = 100 μM and IC_50,DPAP1_ = 1.8 μM vs. IC_50,DPAP3_ >> 100 μM, respectively). Also, several compounds show more than 50-fold selectivity towards FP3 compared to FP2 (Fig 4D and 4E and Tables 1 and 2) such as SMDC153437A (IC_50,FP2_ ~ 100 μM vs. IC_50,FP3_ = 2.3 μM) or some DOPTACN compounds (SMDC170123: IC_50,FP2_ ~ 35 μM vs. IC_50,FP3_ = 0.093 μM; SMDC178801: IC_50,FP2_ = 17 μM vs. IC_50,FP3_ = 0.069 μM; SMDC178822: IC_50,FP2_ = 12 μM vs. IC_50,FP3_ = 0.08 μM).

DOPTACN compounds show partial inhibition of all DPAPs but full inhibition of the falcipains with the exception of FP3 inhibition by SMDC170123 (80% inhibition). The fact that this family of compound achieves different levels of maximum inhibition for different proteases (10–20% for DPAP3, 45–65% for CatC, 60–85% for DPAP1, and 80–100% for the FPs) suggests a conserved allosteric partial inhibition mechanism. Although we observed relatively flat SAR for this compound family against DPAP1, this was not the case for other proteases, thus indicating that these compounds might bind into a specific pocket rather than through a non-specific mechanism (Fig 4 and Tables 1 and 2). Interestingly, this class of compounds is consistently more potent against DPAP3 or CatC than against DPAP1 or the FPs (IC_50_ values), but they achieve lower levels of maximum inhibition. Enzyme dependent effects were also observed for other compounds: SMDC97214 fully inhibits DPAP1 and the FPs but only achieves 80% inhibition of CatC, SMDC153437A fully inhibits DPAP3 and FP3 but only inhibits DPAP1 by 80%; and SMDC103222 inhibits DPAP1, CatC and FP3 by 50, 90, and 100%, respectively.

### Mechanism of inhibition studies

**Fig 5:**
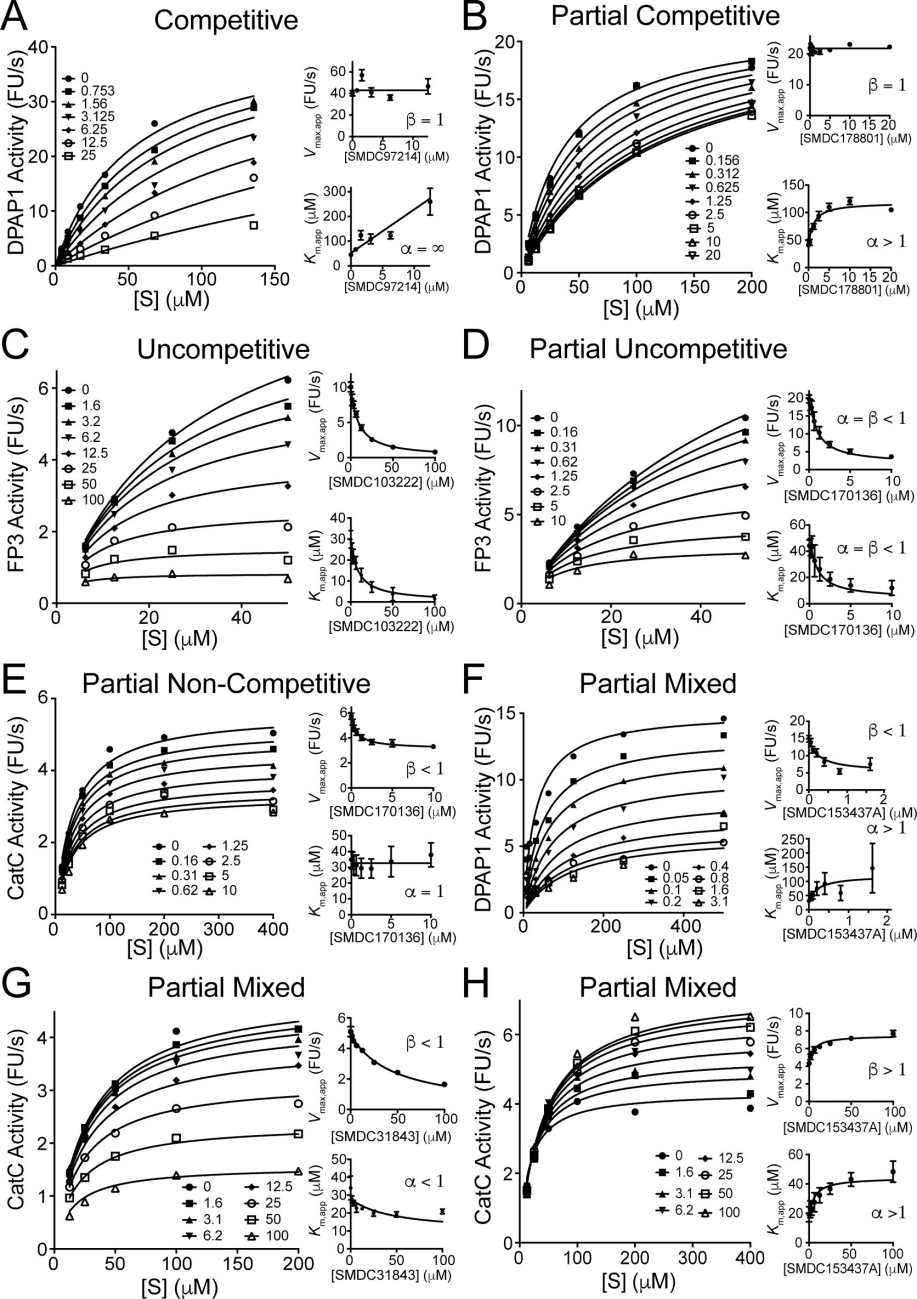
Representative cases of different mechanisms of inhibition. Based on our MOI studies, all compounds inhibit the different proteases tested *via* one of six different inhibition models. A representative data set for each of these MOIs is shown in each panel: (**A**) competitive (Eq 3), (**B**) partial competitive (Eq 6), (**C**) uncompetitive (Eq 4), (**D**) partial uncompetitive (Eq 7), (**E**) partial non-competitive (Eq 8), and (**F-H**) partial mixed inhibition (Eq 9). Three different case of partial mixed inhibition are shown: inhibitor decreases *V*_max,app_ and increases *K*_m,app_ (**F**), inhibitor decreases *V*_max,app_ and *K*_m,app_ (**G**), and inhibitor increases *V*_max,app_ and *K*_m,app_ (**H**). The big graph in each panel shows the global fit of the data set (a matrix of initial velocities containing 4–6 substrate concentrations by 6–8 inhibitor concentrations) to the appropriate inhibition model. Inhibition concentrations in the legend are in micromolar. The small graphs show the independently obtained *K*_m,app_ and *V*_max,app_ values at each inhibitor concentration. The lines in these graphs represent the predicted dependence of *K*_m,app_ and *V*_max,app_ on inhibition concentration calculated based on the inhibition parameters obtained from the global data fit using the equations reported in the S5 Table. Error bars in the small graphs represent the standard error of the fit obtained for *K*_m,app_ and *V*_max,app_.

**Fig 6:**
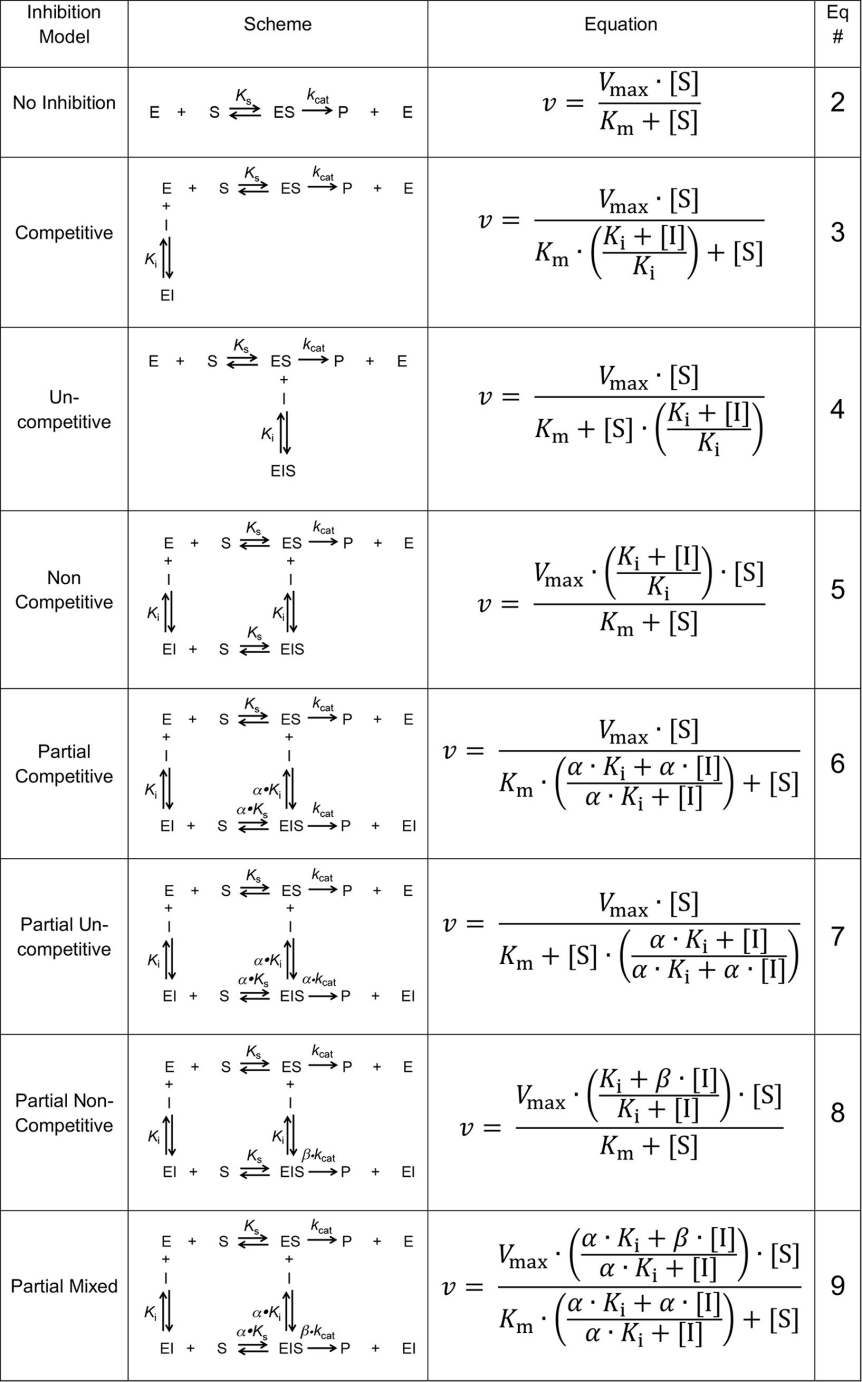
Mechanism of inhibition models. The name of inhibition model, a schematic of the enzyme reaction under steady-state kinetics conditions, and the equation describing each model as a function of *V*_max_, *K*_m_, *K*_i_, *alfa*, and *beta* are shown. *Alfa* is the factor by which *K*_m_ is modified upon inhibitor binding, and *beta* the factor by which *V*_max_ is modified upon inhibitor binding.

To better understand this partial inhibition effect, we performed MOI studies for 12 compounds that showed IC_50_ values below 10 μM for DPAP1. MOI studies for CatC and FP3 were also conducted with selected compounds. For each compound and protease initial turnover rates at different substrate and inhibitor concentrations were measured (Fig 5). Apparent *V*_max_ and *K*_m_ values (*V*_max,app_ and *K*_m,app_) were calculated at each inhibitor concentration (Fig 6, Eq 2). We then examined the dependence of these parameters on inhibitor concentration to determine the appropriate MOI model (Fig 6): full or partial competitive (increase in *K*_m,app_, Fig 6 Eqs 3 and 6, Fig 5A and 5B), full or partial uncompetitive (equal decrease in *V*_max,app_ and *K*_m,app_, Fig 6 Eqs 4 and 7, Fig 5C and 5D), full or partial non-competitive (decrease in *V*_max,app_, Fig 6 Eqs 5 and 8, Fig 5E), or partial mixed inhibition (changes in *V*_max,app_ and *K*_m,app_, Fig 6 Eq 9, Fig 5F–5H). Each data set was then globally fitted to the appropriate MOI model using GraphPad Prism. Fig 5 shows a representative data set for each MOI model. All other MOI fits are shown in S3 Fig. To statistically justify which one of these models better fitted our data, we performed null-hypothesis F-tests using GraphPadPrism as reported in S4 Table. All inhibition parameters (*K*_i_, *α*, and Δ) are reported in Table 3; *α* and *β* indicate the factor by which *K*_m_ and *k*_cat_ are modified at saturating inhibitor concentrations, respectively.

**Table 3:** Mechanism of inhibition studies results.

	DPAP1				CatC				FP3			
SMDC #	Model	*K*_i_ (μM)	*α*	*β*	Model	*K*_i_ (μM)	*α*	*β*	Model	*K*_i_ (μM)	*α*	*β*
112054	COM	0.44 (0.07)	= ∞	0 < *β* < ∞	N.D.				N.D.			
97214	COM	3.0 (0.3)	= ∞	0 < *β* < ∞	N.D.				N.D.			
31843	P-C	6.2 (0.9)	5.6 (0.7)	= 1	P-M	140 (50)	0.33 (0.06)	0 (0.04)	UNC	14.2 (0.1)	N.A.	
103222	P-C	1.1 (0.2)	2.12 (0.09)	= 1	N.D.				UNC	9.1 (0.6)	N.A.	
168313	P-C	4.1 (0.6)	2.8 (0.2)	= 1	N.D.				N.D.			
170136	P-C	1.4 (0.3)	2.3 (0.1)	= 1	P-NC	0.8 (0.2)	= 1	0.56 (0.02)	P-U	12 (4)	0.07 (0.02)	
170156	N.D.				P-NC	3.7 (0.9)	= 1	0.52 (0.04)	N.D.			
178790	P-C	0.40 (0.07)	2.7 (0.2)	= 1	N.D.				N.D.			
178801	P-C	0.36 (0.06)	3.2 (0.2)	= 1	N.D.				P-M	6.6 (2.5)	0.22 (0.09)	0.09 (0.03)
170123	P-M	1.3 (0.4)	4.3 (1.3)	1.4 (0.3)	P-NC	0.95 (0.3)	= 1	0.66 (0.03)	P-M	3.8 (0.9)	0.38 (0.09)	0.23 (0.03)
106517	P-M	1.0 (0.3)	1.3 (0.2)	0.85 (0.05)	N.D.				N.D.			
153437A	P-M	0.06 (0.02)	4.0 (1.5)	0.35 (0.05)	P-M	2.4 (1)	2.6 (0.6)	1.7 (0.1)	N.D.			

Inhibition models: COM, competitive (Eq 3); UNC, uncompetitive (Eq 4); P-M, partial mixed (Eq 9); P-C, partial competitive (Eq 6); P-NC, partial non-competitive (Eq 8); P-U, partial uncompetitive (Eq 7). N.D., Not determined; N.A. Not Applicable. Standard errors of the fit for each parameter are shown in parentheses.

Most compounds showing partial inhibition behavior increased *K*_m,app_ (1.3 < *α* < 5.6) and/or decrease *V*_max,app_ (0.09 < *β* < 0.85). However, for several compounds we observed a 40–70% increase in *V*_max,app_ (*β*> 1 for inhibition of DPAP1 by SMDC170123 or of CatC by SMDC153437A) or a 60–80% decrease in *K*_m,app_ (*α*< 1 for inhibition of CatC by SMDC31843 or of FP3 by SMDC178801 and SMDC170123). This increase in substrate affinity (*K*_m,app_) or maximum turnover rate (*V*_max,app_) upon inhibitor binding provides strong evidence of allosteric effects and raises the possibility that structurally related compounds might act as allosteric activators. Indeed, Fig 5H clearly shows that at saturating concentrations of substrate, SMDC153437A increases the activity of CatC. Finally, inhibition of FP3 by SMDC103222 and SMDC31843 is consistent with an uncompetitive inhibition model, indicating that this compound likely binds the enzyme-substrate complex.

Although structurally related compounds seem to inhibit a specific enzyme through different MOIs, or a single inhibitor inhibits different enzymes via different MOIs, it is important to mention that all inhibition models except uncompetitive inhibition can be explain as particular cases of the partial-mixed inhibition model. The values of the *α* and *β* parameters indicate the factor by which *K*_m_ and *k*_cat_ are modified upon inhibitor binding, respectively (Fig 6). Therefore, for competitive inhibition, *α* = ∞; for partial competitive, *β* = 1; for partial non-competitive, *α* = 1; and for partial uncompetitive, *α* = *β*. Note that allosteric activators would decrease *K*_m_ (*α* < 1) and/or increase *k*_cat_ (*β* > 1).

### Selected CatC inhibitors induce conformational changes independently of active site occupancy

**Fig 7:**
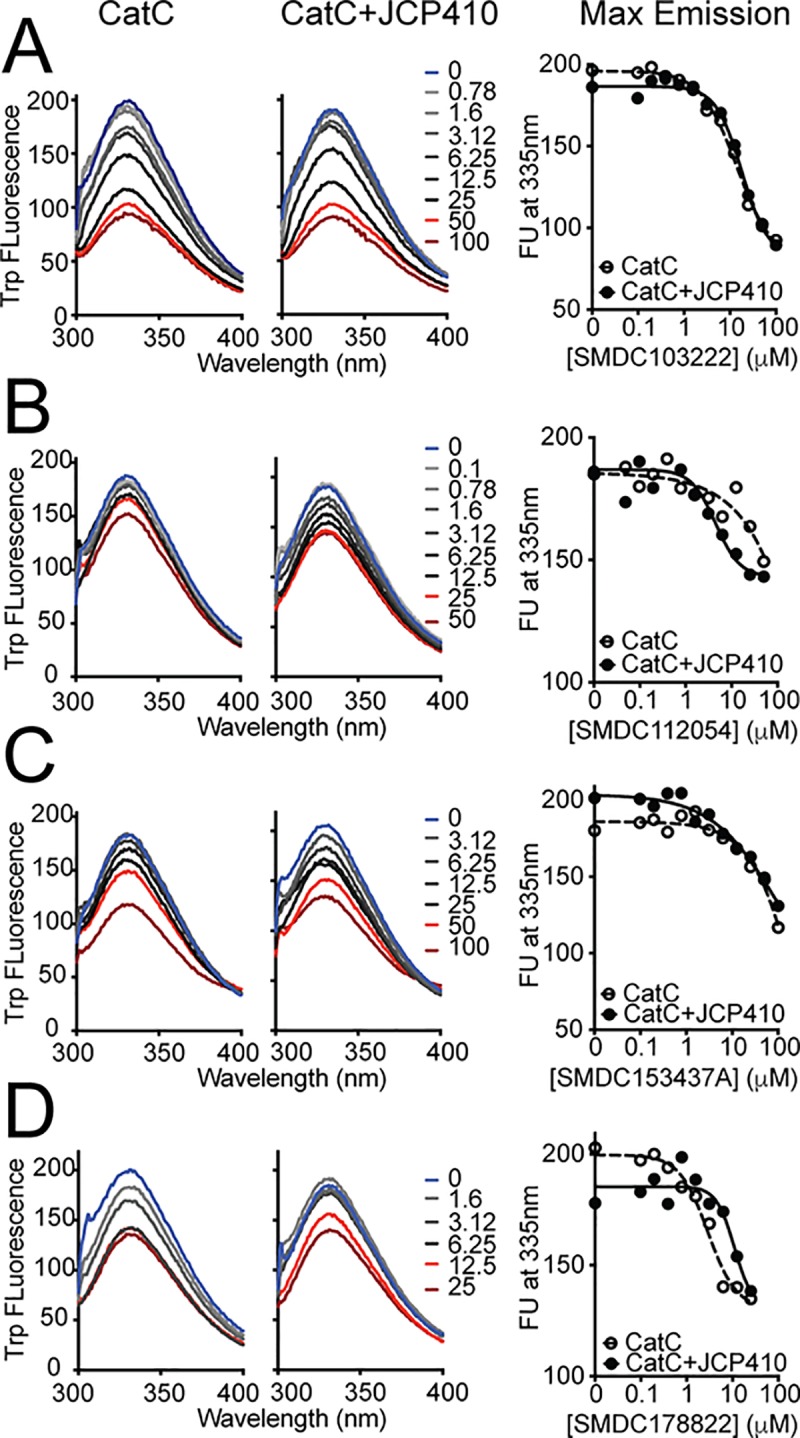
Effect of inhibitor binding on Trp fluorescence emission. The Trp emission spectra (λ_ex_ = 280 nm) obtained after treating 1 μM of CatC with different concentrations of four different inhibitors are shown before (left graphs) and after (middle graph) pre-incubation of CatC with JCP410, a potent covalent inhibitor of CatC. (**A**) SMDC103222, (**B**) SMDC112054, (**C**) SMDC153437A, and (**D**) SMDC178822. In all cases, compound binding decreased Trp fluorescence emission. Dose response curves at the maximum emission wavelength (335 nm) are shown on the right graphs. Inhibitor concentrations in the legend of the emission spectra graphs are in micromolar.

To determine whether some of the identified inhibitors induce a conformational change upon binding, we measured the intrinsic tryptophan fluorescence emission spectra of CatC in the presence of four different inhibitors (SMDC103222, SMDC112054, SMDC153437A, and SMDC178822). As shown in Fig 7, these four compounds decrease the maximum level of Trp fluorescence in a dose dependent manner. More importantly, these changes in Trp fluorescence were also observed in the presence of JCP410, an irreversible inhibitor that covalently modifies the catalytic Cys of CatC. Overall, these results provide further evidence that these compounds do not bind into the active sites of DPAPs, and that they are likely acting through an allosteric mechanism. However, it is important to mention that the fluorogenic substrates or the covalent inhibitor used in our studies do not bind beyond the S2’ pockets. Therefore, we cannot rule out the possibility that some of these compounds might influence substrate turnover by binding into the S3’-S4’ pockets (in which the case, some of these compounds might act as competitive inhibitors of natural substrates).

## Discussion

Our HTS approach in parasite lysates has identified a variety of novel compound scaffolds that inhibit not only DPAPs but also other clan CA cysteine proteases. Surprisingly, most hits identified in this study seem to be allosteric inhibitors. A possible explanation for this result might be the fact that the screen was performed in parasite lysates. DPAPs cleave dipeptides from the free N-terminus of proteins and peptides. Because the screen was performed under acidic conditions, most proteases that are usually found in the digestive vacuole (four aspartyl proteases (plasmepsins), three falcipains, falcilysin (metalloprotease), and several aminopeptidases)[7] were likely active during the course of the assay and would have cleaved a variety of proteins present in the lysate, thus generating a large abundance of free N-termini. These would have likely been recognized and cleaved by DPAP1 since this protease has a broad substrate specificity[35]. Screening compounds in the presence of a large number of competitive substrates might have therefore biased the identification of hits towards inhibitors that do not bind into the substrate binding pocket. To the best of our knowledge, these are the first allosteric DPAP inhibitors reported to date. However, allosteric inhibitors have been previously documented for clan CA proteases. For example, natural products such as heme[36] or suramin[37], and small molecule chalcone inhibitors[38] have been shown to be allosteric inhibitors of the falcipains. Also, allosteric inhibitors have been developed for cathepsin K[39].

There are several advantages in the development of allosteric inhibitors, the most obvious being that the inhibitor does not need to compete with natural substrates in order to bind its target. This might be beneficial for inhibiting DPAP1 since this enzyme is likely working under substrate saturation conditions in the digestive vacuole where a variety of DPAP substrates are being generated continuously by upstream and downstream proteases. Therefore, sustained inhibition of DPAP1 might be better achieved with allosteric inhibitors than with reversible competitive inhibitors. Although the use of partial inhibitors is unlikely to be a viable strategy for antimalarial treatment, the structure of these compounds could be optimized toward 100% inhibition, as is the case for inhibition of FP3 by some of the identified hits. That said, we cannot rule out the possibility that the partial inhibitors identified here might fully inhibit the cleavage of natural substrates if they bind into the S3’-S4’ pockets or into an exosite that might be important for substrate recognition.

Finally, one of the concerns when developing antimalarial drugs is the importance to avoid off-target inhibition of host homologues, in this case CatC. However, *in vivo* studies have shown that full and sustained inhibition of CatC is required to observe a decrease in the activity of neutrophil elastase, which is activated by CatC[40,41]. Therefore, developing inhibitors that partially inhibit CatC but fully inhibit *Plasmodium* DPAPs might be a good strategy to prevent off-target effects due to CatC inhibition.

To the best of our knowledge the compounds identified in this study are the first reported allosteric inhibitors for dipeptidyl aminopeptidases. This opens completely new medicinal chemistry avenues for inhibitor development, either as tools to study the biological function of these enzymes, or as starting points for drug development. However, their poor antiparasitic activity and their cytotoxic effects against human cells at high micromolar concentrations indicate that more potent and specific inhibitors need to be designed to determine whether developing DPAP allosteric inhibitors is a good strategy to generate antiparasitic compounds. Although DPAPs have been shown to play important functions in both *Plasmodium*[7] and *Toxoplasma*[42] parasites, little is known about their molecular function in these organisms or in other unicellular pathogens despite being conserved in most eukaryotic organisms. Therefore, inhibitors developed based on the scaffolds identified in this study might be instrumental in understanding the role of DPAPs in other pathologies such as Chagas’ disease, leishmaniasis, African trypanosomiasis and babesiosis.

## Supporting information

S1 Fig Antiparasitic activity of selected hits. A synchronized culture of *P*. *falciparum* at 1% parasitemia and 1% haematocrit was treated at ring stage with different concentrations of the indicated compounds and cultured for 72 h. Samples were then fixed, the iRBCs stained with propidium iodide, and the parasitemia quantified by flow cytometry. Normalized parasitemia relative to DMSO controls is shown. EC50 values are reported in Tables 1 and 2.
(PDF)

S2 Fig Dose-dependent cytotoxic effects. HFF cells were treated for 24h with different concentrations of selected inhibitors. Cell viability was measured using the CellTiter-Glo Assay (Promega), a biolumniscence-based assay that measures ATP levels within cells. Four biological replicates were performed for this experiment. The averaged normalized luminescence signal was fitted to a dose response curve. The cytotoxic EC50 values are reported in Tables 1 and 2. Error bars represent standard error of the mean.
(PDF)

S3 Fig Mechanisms of inhibition studies not shown in Fig 5. The inhibition model used to globally fit the data is shown above each large graph. As in Fig 5, the *K*_m,app_ and *Vmax*,*app* values obtained at each inhibitor concentration are shown in the small graphs and the lines in these graphs represent the predicted dependence of these parameters based on the *K*_i_, *α* and *β* values obtained from the global data fit (reported in Table 3). For each MOI data set, the inhibitor and enzyme that were investigated can be seen in the X-axis of the small graph and the Y-axis of the large graph, respectively. Inhibitor concentrations in the legend of the large graph are in micromolar. Error bars in the small graphs represent the standard error of the fit obtained for *K*_m,app_ and *V*_max,app_.
(PDF)

S1 Table Purchased compounds summary table. (XLSX)

S2 Table Compounds structure and DPAP1 HTS results. (XLSX)

S3 Table DOPTACN compounds present in the HTS library. (XLSX)

S4 Table F-test results to statistically validate the chosen MOI model. (PDF)

S5 Table Dependence of *V*_max,app_ and *K*_m,app_ on inhibition parameters. (PDF)
