# Supplementary material for: Identification of Plasmodium dipeptidyl aminopeptidase allosteric inhibitors by high throughput screening
Source: PLoS One. 2019 Dec 18;14(12):e0226270. doi: 10.1371/journal.pone.0226270 (PMC6919601; doi:10.1371/journal.pone.0226270)
Supplement: S1 Fig — A synchronized culture of P. falciparum at 1% parasitemia and 1% haematocrit was treated at ring stage with different concentrations of the indicated compounds and cultured for 72 h. Samples were then fixed, the iRBCs stained with propidium iodide, and the parasitemia quantified by flow cytometry. Normalized parasitemia relative to DMSO controls is shown. EC50 values are reported in Tables 1 and 2. (PDF) [file pone.0226270.s001.pdf]

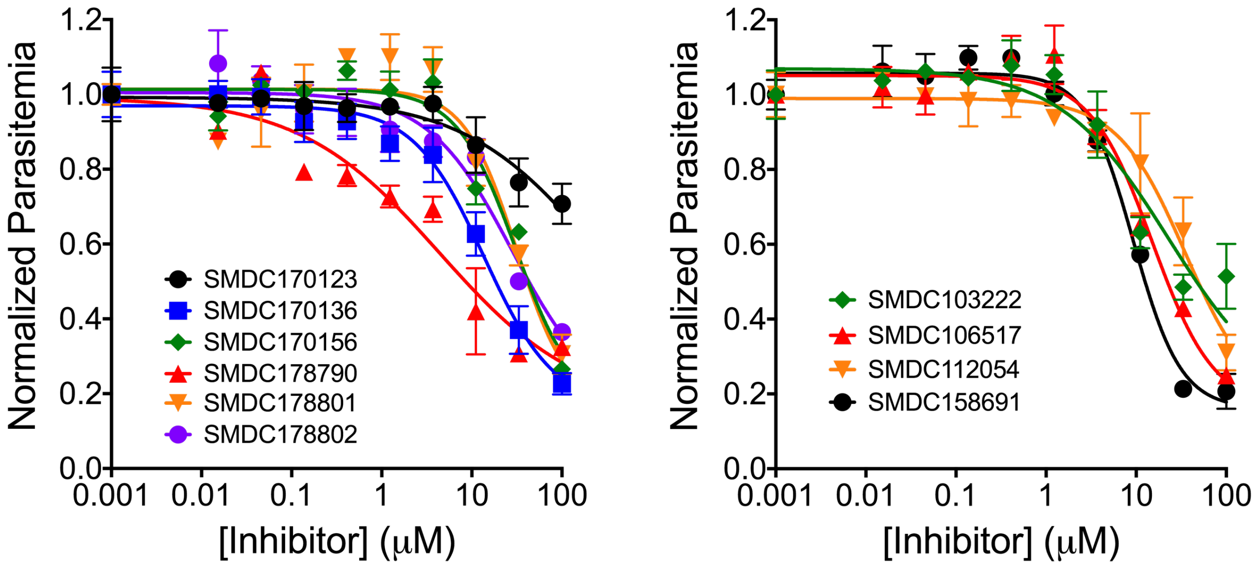

S1 Figure. Antiparasitic activity of selected hits. A synchronized culture of *P. falciparum* at 1% parasitemia and 1% haematocrit was treated at ring stage with different concentrations of the indicated compounds and cultured for 72 h. Samples were then fixed, the iRBCs stained with propidium iodide, and the parasitemia quantified by flow cytometry. Normalized parasitemia relative to DMSO controls is shown. EC_{50} values are reported in Tables 1 and 2.
