# Supplementary material for: Identification of Plasmodium dipeptidyl aminopeptidase allosteric inhibitors by high throughput screening
Source: PLoS One. 2019 Dec 18;14(12):e0226270. doi: 10.1371/journal.pone.0226270 (PMC6919601; doi:10.1371/journal.pone.0226270)
Supplement: S2 Fig — HFF cells were treated for 24h with different concentrations of selected inhibitors. Cell viability was measured using the CellTiter-Glo Assay (Promega), a biolumniscence-based assay that measures ATP levels within cells. Four biological replicates were performed for this experiment. The averaged normalized luminescence signal was fitted to a dose response curve. The cytotoxic EC50 values are reported in Tables 1 and 2. Error bars represent standard error of the mean. (PDF) [file pone.0226270.s002.pdf]

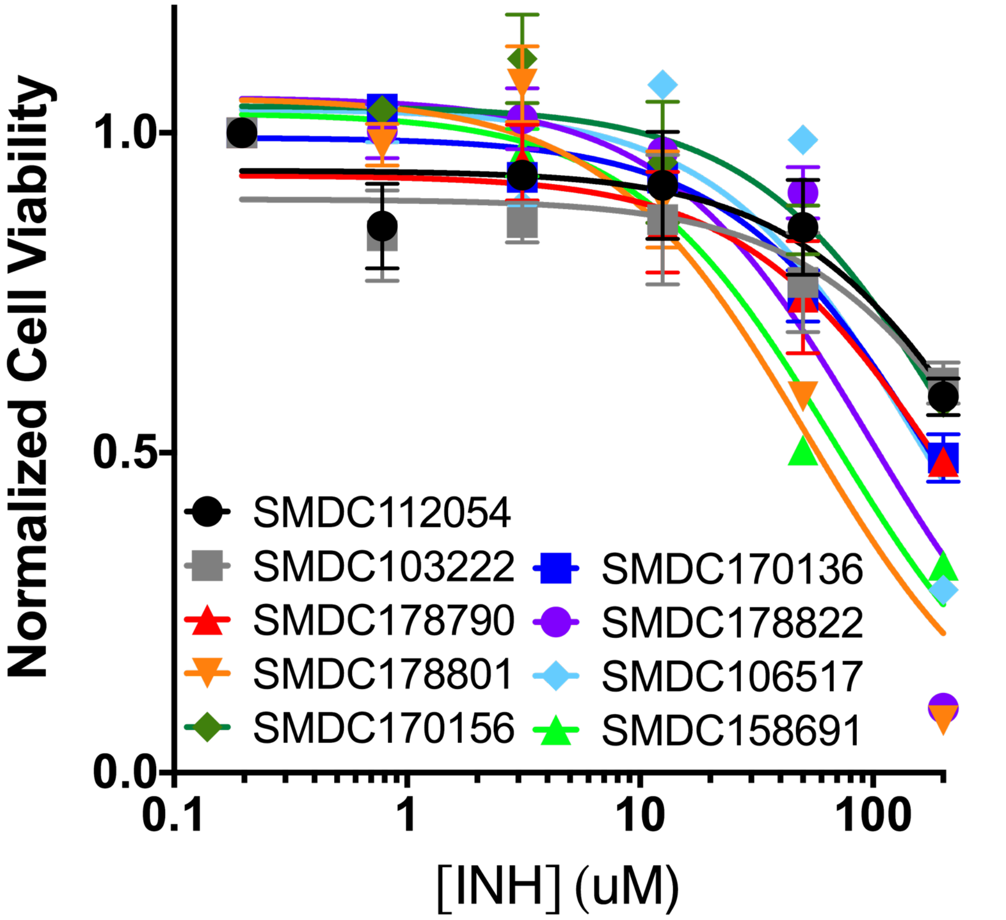


**S2 Figure. Dose-dependent cytotoxic effects.** HFF cells were treated for 24h with different concentrations of selected inhibitors. Cell viability was measured using the CellTiter-Glo Assay (Promega), a biolumniscence-based assay that measures ATP levels within cells. Four biological replicates were performed for this experiment. The averaged normalized luminescence signal was fitted to a dose response curve. The cytotoxic $EC_{50}$ values are reported in Tables 2 & 3. Error bars represent standard error of the mean.
