# Supplementary material for: Identification of Plasmodium dipeptidyl aminopeptidase allosteric inhibitors by high throughput screening
Source: PLoS One. 2019 Dec 18;14(12):e0226270. doi: 10.1371/journal.pone.0226270 (PMC6919601; doi:10.1371/journal.pone.0226270)
Supplement: S3 Fig — The inhibition model used to globally fit the data is shown above each large graph. As in Fig 5, the Km,app and Vmax,app values obtained at each inhibitor concentration are shown in the small graphs and the lines in these graphs represent the predicted dependence of these parameters based on the Ki, α and β values obtained from the global data fit (reported in Table 3). For each MOI data set, the inhibitor and enzyme that were investigated can be seen in the X-axis of the small graph and the Y-axis of the large graph, respectively. Inhibitor concentrations in the legend of the large graph are in micromolar. Error bars in the small graphs represent the standard error of the fit obtained for Km,app and Vmax,app. (PDF) [file pone.0226270.s003.pdf]

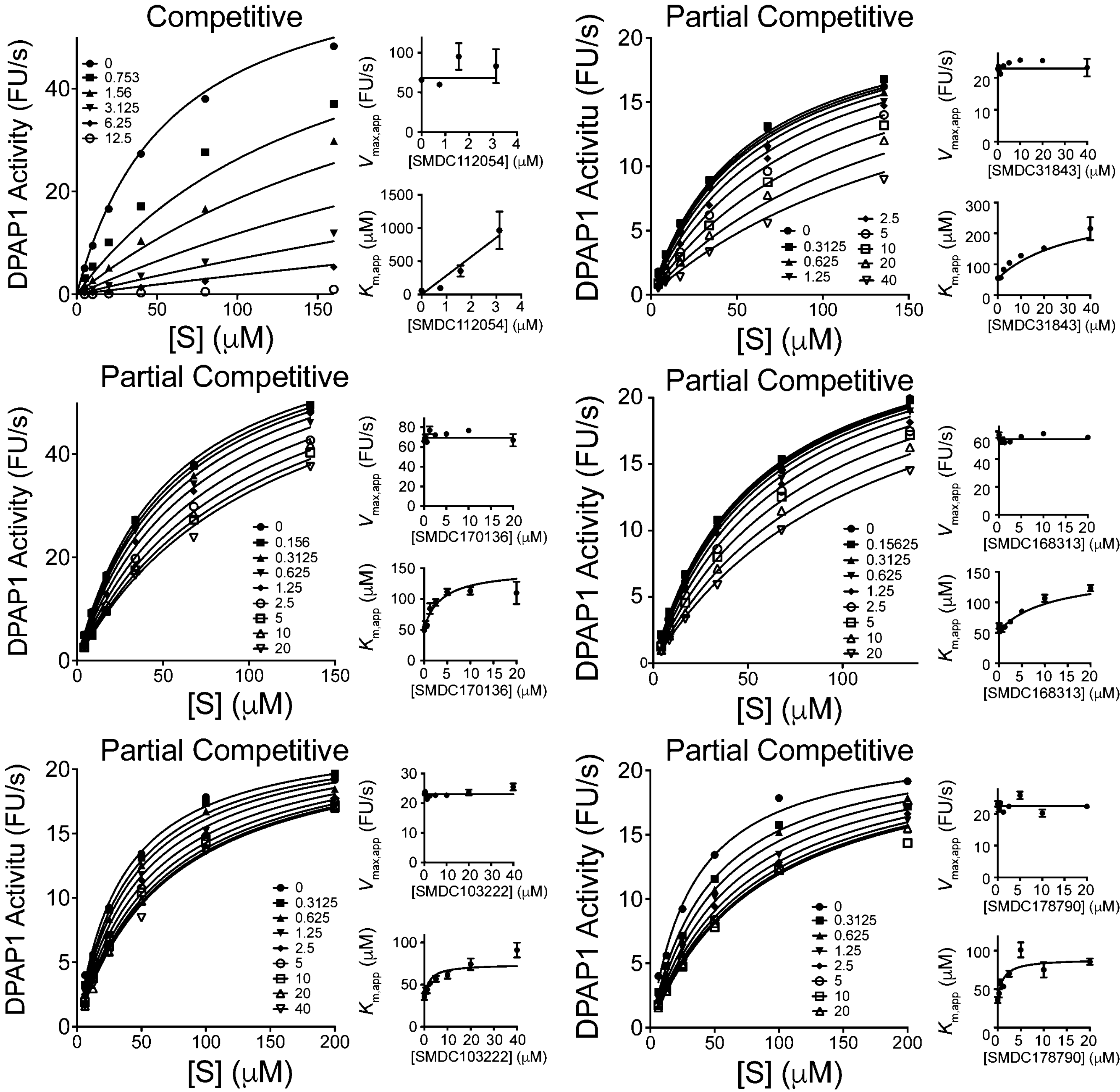

Competitive
DPAP1 Activity (FU/s)
[S] (μM)
0
0.753
1.56
3.125
6.25
12.5
$V_{max,app}$ (FU/s)
[SMDC112054] (μM)
$K_{m,app}$ (μM)
[SMDC112054] (μM)
Partial Competitive
DPAP1 Activitu (FU/s)
[S] (μM)
0
0.3125
0.625
1.25
2.5
5
10
20
40
$V_{max,app}$ (FU/s)
[SMDC31843] (μM)
$K_{m,app}$ (μM)
[SMDC31843] (μM)
Partial Competitive
DPAP1 Activity (FU/s)
[S] (μM)
0
0.156
0.3125
0.625
1.25
2.5
5
10
20
$V_{max,app}$ (FU/s)
[SMDC170136] (μM)
$K_{m,app}$ (μM)
[SMDC170136] (μM)
Partial Competitive
DPAP1 Activity (FU/s)
[S] (μM)
0
0.15625
0.3125
0.625
1.25
2.5
5
10
20
$V_{max,app}$ (FU/s)
[SMDC168313] (μM)
$K_{m,app}$ (μM)
[SMDC168313] (μM)
Partial Competitive
DPAP1 Activitu (FU/s)
[S] (μM)
0
0.3125
0.625
1.25
2.5
5
10
20
40
$V_{max,app}$ (FU/s)
[SMDC103222] (μM)
$K_{m,app}$ (μM)
[SMDC103222] (μM)
Partial Competitive
DPAP1 Activity (FU/s)
[S] (μM)
0
0.3125
0.625
1.25
2.5
5
10
20
$V_{max,app}$ (FU/s)
[SMDC178790] (μM)
$K_{m,app}$ (μM)
[SMDC178790] (μM)

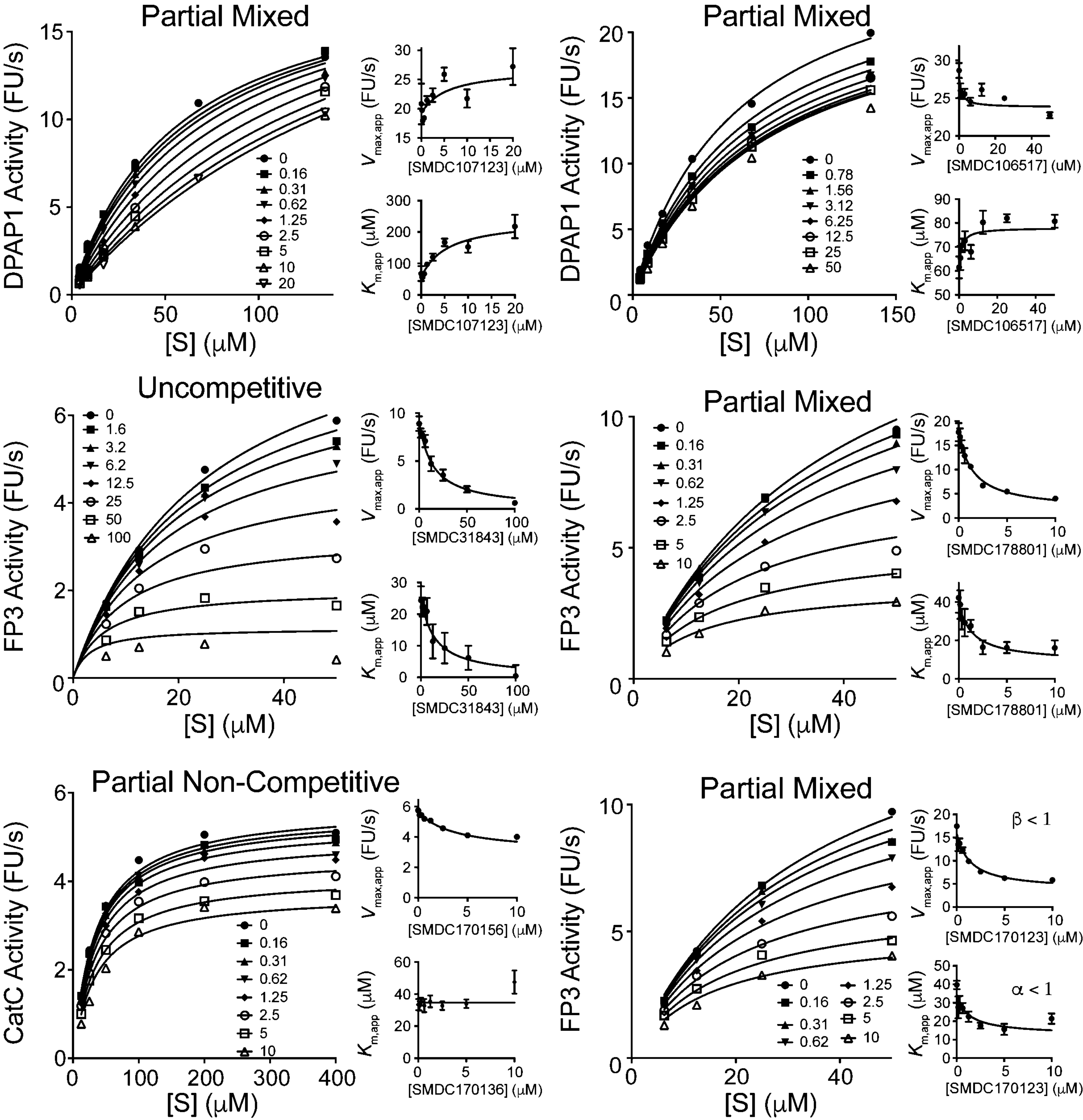

Partial Mixed
DPAP1 Activity (FU/s)
[S] (μM)
0
0.16
0.31
0.62
1.25
2.5
5
10
20
$V_{max,app}$ (FU/s)
[SMDC107123] (μM)
$K_{m,app}$ (μM)
[SMDC107123] (μM)
Partial Mixed
DPAP1 Activity (FU/s)
[S] (μM)
0
0.78
1.56
3.12
6.25
12.5
25
50
$V_{max,app}$ (FU/s)
[SMDC106517] (μM)
$K_{m,app}$ (μM)
[SMDC106517] (μM)
Uncompetitive
FP3 Activity (FU/s)
[S] (μM)
0
1.6
3.2
6.2
12.5
25
50
100
$V_{max,app}$ (FU/s)
[SMDC31843] (μM)
$K_{m,app}$ (μM)
[SMDC31843] (μM)
Partial Mixed
FP3 Activity (FU/s)
[S] (μM)
0
0.16
0.31
0.62
1.25
2.5
5
10
$V_{max,app}$ (FU/s)
[SMDC178801] (μM)
$K_{m,app}$ (μM)
[SMDC178801] (μM)
Partial Non-Competitive
CatC Activity (FU/s)
[S] (μM)
0
0.16
0.31
0.62
1.25
2.5
5
10
$V_{max,app}$ (FU/s)
[SMDC170156] (μM)
$K_{m,app}$ (μM)
[SMDC170136] (μM)
Partial Mixed
FP3 Activity (FU/s)
[S] (μM)
0
0.16
0.31
0.62
1.25
2.5
5
10
$V_{max,app}$ (FU/s)
[SMDC170123] (μM)
β < 1
$K_{m,app}$ (μM)
[SMDC170123] (μM)
α < 1

S2 figure. Mechanisms of inhibition studies not shown in Fig 3. The inhibition model used to globally fit the data is shown above each large graph. As in Fig 3, the $K_{m,app}$ and $V_{max,app}$ values obtained at each inhibitor concentration are shown in the small graphs and the lines in these graphs represent the predicted dependence of these parameters based on the K_i, *a* and *b* values obtained from the global data fit (reported in Table 4). For each MOI data set, the inhibitor and enzyme that were investigated can be seen in the X-axis of the small graph and the Y-axis of the large graph, respectively. Inhibitor concentrations in the legend of the large graph are in micromolar. Error bars in the small graphs represent the standard error of the fit obtained for $K_{m,app}$ and $V_{max,app.}$
