# Supplementary material for: Identification of Plasmodium dipeptidyl aminopeptidase allosteric inhibitors by high throughput screening
Source: PLoS One. 2019 Dec 18;14(12):e0226270. doi: 10.1371/journal.pone.0226270 (PMC6919601; doi:10.1371/journal.pone.0226270)
Supplement: S4 Table — (PDF) [file pone.0226270.s007.pdf]

S4 Table. F-test results to statistically validate the chosen MOI model.

	DPAP1				CatC				FP3			
Compound	Model	Null Hyp Model	p-value	Null Hyp Answer	Model	Null Hyp Model	p-value	Null Hyp Answer	Model	Null Hyp Model	p-value	Null Hyp Answer
SMDC170123	P-M	**P-C**	0.1	Accept	P-M	**P-NC**	0.3	Accept	**P-M**	P-U	0.006	Reject
SMDC170136	P-M	**P-C**	0.1	Accept	P-M	**P-NC**	0.7	Accept	P-M	**P-U**	0.1	Accept
SMDC170156					P-M	**P-NC**	0.09	Accept				
SMDC178790	P-M	**P-C**	0.5	Accept								
SMDC178801	P-M	**P-C**	0.5	Accept					**P-M**	P-U	0.03	Reject
SMDC170123	**P-M**	P-C	0.03	Reject								
SMDC31843	P-M	**P-C**	0.1	Accept	P-M	**P-M; β=0**	N.C.	Accept	**UNC**	P-U	N.C.	Accept
SMDC103222	P-M	**P-C**	0.7	Accept					**UNC**	P-U	N.C.	Accept
SMDC168313	P-M	**P-C**	0.5	Accept								
SMDC106517	**P-M**	P-C	0.005	Reject								
SMDC153437A	**P-M**	P-C	0.002	Reject	**P-M**	P-U	0.01	Reject				

The chosen inhibitor model after performing the F-test is highlighted in bold. The null hypothesis was rejected (i.e. the data were fitted to the model that has an additional parameter) if the p-value was below 0.05. Abbreviations of inhibition models: P-M, partial mixed; P-C, partial competitive; UNC, uncompetitive; and P-U, partial uncompetitive.
