# Supplementary material for: Identification of Plasmodium dipeptidyl aminopeptidase allosteric inhibitors by high throughput screening
Source: PLoS One. 2019 Dec 18;14(12):e0226270. doi: 10.1371/journal.pone.0226270 (PMC6919601; doi:10.1371/journal.pone.0226270)
Supplement: S5 Table — (PDF) [file pone.0226270.s008.pdf]

S5 Table. Dependence of $V_{max,app}$ and $K_{m,app}$ on inhibition parameters.

Inhibition Model	Equation in Table 3	$V_{max,app}$	$K_{m,app}$
Competitive	$v = \frac{V_{max} \cdot [S]}{K_m \cdot \left(\frac{K_i + [I]}{K_i}\right) + [S]}$	$V_{max,app} = V_{max}$	$K_{max,app} = K_m \left(1 + \frac{[I]}{K_i}\right)$
Un-competitive	$v = \frac{V_{max} \cdot [S]}{K_m + [S] \cdot \left(\frac{K_i + [I]}{K_i}\right)}$	$V_{max,app} = V_{max} \cdot \left(\frac{K_i}{K_i + [I]}\right)$	$K_{max,app} = K_m \cdot \left(\frac{K_i}{K_i + [I]}\right)$
Partial Competitive	$v = \frac{V_{max} \cdot [S]}{K_m \cdot \left(\frac{\alpha \cdot K_i + \alpha \cdot [I]}{\alpha \cdot K_i + [I]}\right) + [S]}$	$V_{max,app} = V_{max}$	$K_{max,app} = K_m \left(\frac{K_i + [I]}{K_i + \frac{[I]}{\alpha}}\right)$
Partial Non-Competitive	$v = \frac{V_{max} \cdot \left(\frac{K_i + \beta \cdot [I]}{K_i + [I]}\right) \cdot [S]}{K_m + [S]}$	$V_{max,app} = V_{max} \cdot \left(\frac{K_i + \beta \cdot [I]}{K_i + [I]}\right)$	$K_{m,app} = K_m$
Partial Un-competitive	$v = \frac{V_{max} \cdot [S]}{K_m + [S] \cdot \left(\frac{\alpha \cdot K_i + [I]}{\alpha \cdot K_i + \alpha \cdot [I]}\right)}$	$V_{max,app} = V_{max} \left(\frac{K_i + [I]}{K_i + \frac{[I]}{\alpha}}\right)$	$K_{m,app} = K_m \left(\frac{K_i + [I]}{K_i + \frac{[I]}{\alpha}}\right)$
Partial Mixed	$v = \frac{V_{max} \cdot \left(\frac{\alpha \cdot K_i + \beta \cdot [I]}{\alpha \cdot K_i + [I]}\right) \cdot [S]}{K_m \cdot \left(\frac{\alpha \cdot K_i + \alpha \cdot [I]}{\alpha \cdot K_i + [I]}\right) + [S]}$	$V_{max,app} = V_{max} \cdot \left(\frac{\alpha \cdot K_i + \beta \cdot [I]}{\alpha \cdot K_i + [I]}\right)$	$K_{m,app} = K_m \cdot \left(\frac{K_i + [I]}{K_i + \frac{[I]}{\alpha}}\right)$
